# Comparative Study of Free Radical Grafting and Alkaline Conjugation for Enhanced Resveratrol Incorporation and Whey Protein Functionalities

**DOI:** 10.3390/foods14152596

**Published:** 2025-07-24

**Authors:** Tanaporn Manochai, Suthaphat Kamthai, Thanyaporn Siriwoharn

**Affiliations:** 1 Division of Food Science and Technology, Faculty of Agro-Industry, Chiang Mai University, Chiang Mai 50100, Thailand; bumbimtnp@gmail.com; 2 Division of Packaging Technology, Faculty of Agro-Industry, Chiang Mai University, Chiang Mai 50100, Thailand; suthaphat.k@cmu.ac.th; 3 Lanna Rice Research Center, Chiang Mai University, Chiang Mai 50100, Thailand

**Keywords:** stilbenoid, covalent bonding, protein–polyphenol conjugates, phytochemicals, secondary structure, solubility, emulsifying capacity, foaming capacity, antioxidant activity

## Abstract

Incorporating health-promoting resveratrol into food products is challenging, primarily due to its poor solubility. Covalent conjugation is a promising, low-energy, and environmentally friendly strategy to overcome this limitation. This study compared the effectiveness of free radical grafting and alkaline methods for covalently conjugating whey protein isolate (WPI) with resveratrol. Conjugates were evaluated for molecular weight, structural characteristics, functional properties, and antioxidant activities. Both methods yielded conjugates with enhanced solubility relative to native resveratrol, with fold increases from 7.6 to 21.7 for the free radical grafting and from 8.1 to 23.6 for the alkaline method. Conjugates prepared via free radical grafting exhibited greater increases in molecular weight (10–100 kDa range), higher resveratrol incorporation (up to 17.6%), and superior functional properties compared to the alkaline conjugates (*p* < 0.05). Specifically, emulsifying activity, foaming capacity, and foaming stability improved by up to 64.7%, 45.8%, and 220.9%, respectively, compared to WPI. The antioxidant activities of the free radical grafting conjugates were 1.3- to 3.6-fold higher than those of alkaline conjugates. These findings highlight free radical grafting of WPI as a promising approach for incorporating resveratrol and improving the functionality of protein-based ingredients in functional food products.

## 1. Introduction

Resveratrol is a well-studied phytochemical with established antioxidant and anti-inflammatory effects against chronic diseases such as diabetes, obesity, cancer, and cardiovascular disorders [1,2]. However, its application in functional foods is hindered by poor water solubility, resulting in low stability and bioavailability [2,3,4]. Bioactive compounds with low solubility typically face a major challenge in delivering sufficient concentrations, particularly in aqueous-based food and beverage products, to confer health benefits [5]. Although encapsulation and nanoparticle delivery systems have succeeded in pharmaceutical contexts [6,7,8], their high cost and operational complexity limit their adoption in large-scale food processing [9]. Food-grade covalent conjugation of resveratrol to proteins is a promising, low-energy, and sustainable alternative; however, systematic comparisons of feasible conjugation methods and comprehensive functional evaluations remain lacking.

To address this gap, we directly compared two practical chemical methods—free radical grafting and alkaline treatment—for covalent conjugation of resveratrol with whey protein isolate (WPI). These methods were chosen for their operational simplicity, scalability, and low energy requirements. The free radical grafting method utilizes controlled radicals to activate amino acids on protein chains, facilitating strong phenolic bonds [10], while the alkaline method generates reactive quinones from phenolics to form Schiff base linkages with proteins [11]. WPI was selected due to its high content of conjugatable amino acids [12,13], excellent solubility, and established nutritional benefits [14,15,16].

Previous studies indicate that covalently linking phenolic compounds to proteins can modify protein functionality and confer antioxidant activity, with outcomes dependent on both the phenolic structure and the conjugation approach [10]. However, studies of resveratrol–protein covalent conjugation remain limited, with existing research focusing primarily on the emulsification capacity and antioxidant activities of resveratrol–zein systems [17]. To our knowledge, this is the first study to systematically evaluate the impact of conjugation method on grafting efficiency, structural changes, solubility, and key functional and antioxidant properties of resveratrol–protein conjugates. Our findings provide a practical pathway for expanding resveratrol use in functional foods by overcoming current incorporation barriers through sustainable protein conjugation.

The objective of this study was to identify the most effective strategy for producing WPI–resveratrol conjugates with improved solubility and functional performance.

## 2. Materials and Methods

### 2.1. Materials

Whey protein isolate (WPI, purity 88.53%) and resveratrol (purity > 98%) were purchased from Scidict Plus Co., Ltd. (Bangkok, Thailand). Analytical-grade chemicals and standards used in the experiments were obtained from RCI Labscan Limited (Bangkok, Thailand) and Sigma-Aldrich (St. Louis, MO, USA). The SDS-PAGE protein marker was purchased from Bio-Rad Laboratories (Hercules, CA, USA).

### 2.2. Preparation of WPI–Resveratrol Conjugates

Free radical grafting conjugates were prepared according to Fan et al. [18]. One gram (1 g) of WPI was dispersed in 188 mL of deionized water. Then, 0.25 g of ascorbic acid and 2 mL of hydrogen peroxide (1.0 mM) were added, and the mixture was stirred for 30 min at room temperature. Subsequently, 10 mL of resveratrol solution (containing either 1, 5, or 10 g resveratrol dissolved in 10 mL of 80% (*v*/*v*) ethanol) was added to the mixture and stirred for 24 h. Unreacted resveratrol was removed through centrifugation at 5000 rpm for 15 min using 1 kDa Amicon Ultra centrifugal filters (Merck Millipore, Darmstadt, Germany) and a Z206-A centrifuge (Hermle, Wehingen, Germany). The resulting solution was lyophilized using a FreeZone 4.5 L freeze dryer (Labconco, Kansas City, MO, USA) and stored in aluminum foil bags at −18 °C until analysis.

For alkaline conjugate preparation, the method of Jiang et al. [19] was followed. One gram (1 g) of WPI was dispersed in 190 mL sodium phosphate buffer (0.1 M, pH 9), stirred for 30 min, and then mixed with 10 mL resveratrol solution (1, 5, or 10 g in 10 mL 80% (*v*/*v*) ethanol). After stirring for 24 h, unreacted resveratrol was removed using the same filtration and centrifugation steps. The final solution was lyophilized and stored in an aluminum foil bag at −18 °C until further analysis.

### 2.3. Determination of Total Phenolic Content and % Grafting Efficiency

Determination of total phenolic content was performed according to Liu et al. [20]. Briefly, 0.5 mL of the sample solution (1 mg/mL) was mixed with 2.5 mL of 10% Folin-Ciocalteu reagent and kept in the dark for 5 min. Subsequently, 2 mL of 7.5% (*w*/*v*) sodium carbonate was added. The mixture was vortexed and kept in the dark for 2 h. Absorbance was measured at 760 nm using a microplate reader (Spark^®^ Multimode, Tecan Trading AG, Männedorf, Switzerland). The total phenolic content (*C*, mg gallic acid/g) was calculated using the following equation:

(1) $$C=\frac{C_{1}\times V}{m},$$

where *C*_1_ is the concentration of gallic acid obtained from the standard curve (mg/mL), *V* is the volume of the sample (mL), and *m* is the weight of the sample (g).

Grafting efficiency (%) was calculated as follows:

(2) $$Grafting\;efficiency=\left(\frac{C}{C_{2}}\right)\times 100,$$

where *C* is the total phenolic content in the WPI–resveratrol conjugate (mg/g), and *C_2_* is the total phenolic content in the WPI–resveratrol conjugate before the removal of unreacted resveratrol.

### 2.4. Determination of Molecular Weight (M.W.)

#### 2.4.1. Sodium Dodecyl Sulfate-Polyacrylamide Gel Electrophoresis (SDS-PAGE)

SDS-PAGE was performed using a Mini-Protean Tetra Cell system (Bio-Rad Laboratories) as described by Schägger [21], with minor modifications. Sample solutions (10 mg/mL) were diluted 1:1 with 2× Laemmli sample buffer (950 µL) containing 50 µL of 2-mercaptoethanol. The diluted sample (10 µL) was loaded into each well of the gel, which consisted of a 4% stacking gel and a 15% resolving gel. Electrophoresis was conducted at 120 V for 60 min at room temperature. Gels were stained with Coomassie Blue G-250, destained, and scanned. Molecular weights were determined using Precision Plus Protein Dual Xtra Standards (Bio-Rad Laboratories, Hercules, CA, USA).

#### 2.4.2. High-Performance Size Exclusion Chromatography (HPSEC)

Analysis was performed according to Phongphisutthinant et al. [22] using a high-performance liquid chromatography (HPLC) system (Shimadzu, Kyoto, Japan) equipped with an SRT-C SEC-300 column (5 μm, 7.8 × 300 mm, Sepax Technologies, Newark, DE, USA) and a UV–VIS detector set at 280 nm. Samples were prepared by dissolving 10 mg of sample in 1 mL of distilled water and then filtering the solution through a 0.45 μm membrane filter before injection. An injection volume of 15 μL was used, with a flow rate of 1.0 mL/min and 1 M sodium phosphate buffer (pH 7.0) as the mobile phase. Protein molecular weights were estimated with a calibration curve constructed using standard proteins: *p*-aminobenzoic acid (137 Da), ribonuclease A (13.7 kDa), albumin (44.3 kDa), γ-globulin (150 kDa), and thyroglobulin bovine (640 kDa).

### 2.5. Structural Analysis

#### 2.5.1. UV Spectroscopy

UV absorption spectra of the samples were measured using a microplate reader. Ten milligrams (10 mg) of sample were dissolved in 10 mL of distilled water, followed by a tenfold dilution. The absorbance spectrum was recorded from 200 to 400 nm.

#### 2.5.2. Fourier Transform Infrared Spectrometer (FTIR)

FTIR spectra were obtained following the method of Andrade et al. [23]. Two milligrams (2 mg) of sample were mixed with 200 mg of KBr powder, then pressed into a pellet. Spectra were recorded from 4000 to 400 cm^−1^ with a resolution of 4 cm^−1^ and 64 scans, using a Tensor 27 FTIR Spectrometer (Bruker, Ettlingen, Germany). Secondary structure analysis (α-helix, β-sheet, β-turn, and random coil) was performed by Gaussian curve fitting using OriginPro 8.5.1 (OriginLab Corporation, Northampton, MA, USA).

### 2.6. Determination of Solubility and Functional Properties

#### 2.6.1. Water Solubility

The water solubility of the sample was determined using the method of Rui et al. [24] with minor modifications. Briefly, 0.1 g of sample was dissolved in 10 mL of distilled water, and the pH was adjusted to selected values ranging from pH 3 to pH 9 using 0.1 M HCl or NaOH. The suspension was stirred for 30 min, then centrifuged at 5000 rpm for 15 min at room temperature. The supernatant was collected and lyophilized. Water solubility was calculated using the following equation:

(3) $$\%Water\;solubility=\frac{weight\;of\;freeze\;dried\;sample}{initial\;sample\;weight}\times 100.$$

#### 2.6.2. Emulsifying Properties

The emulsifying properties were determined following the method of Sui et al. [25] with minor modifications. Briefly, 15 mL of sample solution (1 mg/mL, pH adjusted to the selected values) was mixed with 5 mL of soybean oil and homogenized at 10,000 rpm for 1 min using a homogenizer (T25 Digital Ultra TURRAX, IKA, Staufen, Germany). Then, 50 µL of the resulting emulsion was diluted into 5 mL of 0.1% SDS solution. Absorbance at 500 nm was measured immediately (*A*_0_) and after 10 min (*A*_10_) at room temperature using a microplate reader. The emulsifying activity index (EAI) and emulsion stability index (ESI) were calculated as follows:

(4) $$EAI\left(m^{2}|/|g\right)=\frac{2\times 2.303\times A_{0}\times 100}{1.25\times 0.25\times 0.01},\;\mathrm{and}$$

(5)
ESI(%)=A
1
0×100
A
0.

#### 2.6.3. Foaming Properties

The foaming properties were determined according to the method of Hamdani et al. [26] with minor modifications. Fifty milliliters (50 mL) of sample solution (1 mg/mL) were adjusted to the selected pH values (pH 3–9) using 0.1 M HCl or NaOH, and homogenized with a homogenizer at 10,000 rpm for 1 min. Foam volumes were measured in graduated cylinders. Foaming capacity (FC) was calculated as follows:

(6) $$FC(\%)=\frac{Volume\;after\;homogenizing-Volume\;before\;homogenizing}{Volume\;before\;homogenizing}\times 100.$$

The foaming stability (FS) of the sample was determined as the volume of foam remaining after 30 min, calculated as follows:

(7) $$FS(\%)=\frac{Foam\;volume\;after\;30\;min}{Initial\;foam\;volume}\times 100.$$

### 2.7. Determination of Antioxidant Activities

#### 2.7.1. DPPH Radical Scavenging Assay

The DPPH radical scavenging activity was determined according to the method of Liu et al. [27] with minor modifications. Briefly, 25 μL of sample solution (1 mg/mL) was mixed with 200 μL DPPH solution (0.1 mmol/L in methanol) in a 96-well microplate and incubated in the dark at room temperature for 30 min. Absorbance was measured at 517 nm using a microplate reader. Methanol was used as a blank. The scavenging activity of the sample was quantified using a Trolox standard curve and expressed as µmol Trolox equivalent/g sample (µmol TE/g).

#### 2.7.2. ABTS Radical Scavenging Assay

The ABTS radical scavenging activity was determined following the method of Re et al. [28], with minor modifications. The ABTS reagent was prepared by mixing 7 mmol/L ABTS solution with 2.45 mM/L potassium persulfate in deionized water at a 1:1 ratio and incubating the mixture in the dark for 16 h at room temperature. The ABTS reagent was diluted with distilled water to obtain an absorbance of 0.70 ± 0.02 at 734 nm. For the assay, 10 μL of sample solution (1 mg/mL) was added to 240 μL of the diluted ABTS reagent in a 96-well microplate and incubated in the dark at room temperature for 6 min. The absorbance was measured at 734 nm using a microplate reader, with distilled water as the blank. The scavenging activity was calculated using a Trolox standard curve and expressed as µmol TE/g.

#### 2.7.3. FRAP Assay

The FRAP assay was modified from Rui et al. [29]. The FRAP reagent was prepared by mixing acetate buffer (300 mM, pH 3.6), 20 mM ferric chloride hexahydrate solution, and 10 mM 2,4,6-Tris (2-pyridyl)-s-triazine (TPTZ) solution in a 10:1:1 (*v*/*v*/*v*) ratio. Twenty-five microliters (25 μL) of sample solution (1 mg/mL) were added to 200 μL of the FRAP reagent in a 96-well microplate and incubated in the dark at room temperature for 8 min. Absorbance was measured at 593 nm using a microplate reader, with acetate buffer used as the blank. The FRAP value was calculated using a Trolox standard curve and expressed as µmol TE/g.

### 2.8. Statistical Analysis

All experiments were carried out in triplicate and expressed as mean ± standard deviation (SD). Statistical analysis was performed using SPSS software version 17.0 (SPSS Inc., Chicago, IL, USA). One-way analysis of variance (ANOVA) and Duncan’s multiple range test were conducted, with the significance level set at *p* < 0.05.

## 3. Results and Discussion

### 3.1. Structural Characteristics

#### 3.1.1. SDS-PAGE and M.W. Distribution

**Figure 1:**
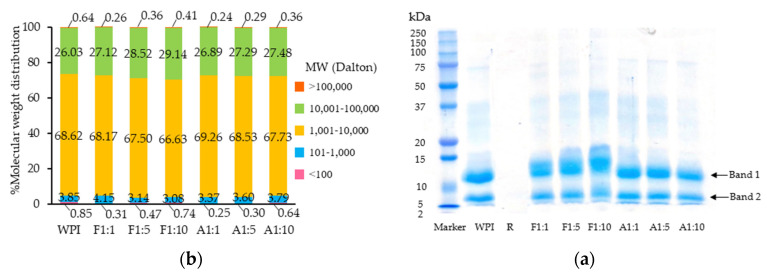
(**a**) SDS-PAGE profiles; (**b**) Molecular weight distribution of whey protein isolate (WPI), resveratrol (R), and WPI–R conjugates prepared using free radical grafting (F) and alkaline (A) methods at WPI/R ratios of 1:1, 1:5, and 1:10.

The SDS-PAGE profile of WPI revealed two major bands (Figure 1a). The first band had an M.W. consistent with α–lactalbumin (α-La, 14.2 kDa), corroborating previous findings [18,30]. The second prominent band (6–8 kDa) likely represents smaller protein components, though its precise identity requires further investigation, potentially via mass spectrometry. Bands corresponding to β-lactoglobulin (β-Lg, 18.2 kDa) and bovine serum albumin (BSA, 66.0 kDa) appeared less intense compared to previous reports [30,31], possibly due to variations in processing methods and thermal pretreatments during WPI production, which can induce protein denaturation or aggregation and thereby affect band visibility [32,33].

The conjugation method and protein-to-resveratrol ratio influenced the SDS-PAGE profiles of the resulting conjugates. Specifically, the free radical grafting method caused bands corresponding to α-La and β-Lg to shift towards higher M.W., and their intensity increased with higher resveratrol concentration (Figure 1a). In contrast, the alkaline method produced less pronounced changes, with minimal shifts or intensity changes observed across different resveratrol concentrations. This pattern is consistent with previous studies showing that α-La and β-Lg are the principal conjugation sites in whey proteins [34].

Quantitative M.W. distribution analysis using HPSEC (Figure 1b and Table S1) confirmed an increased proportion of proteins in the 10–100 kDa range for the free radical grafting conjugates (27.12–29.14%) compared to the alkaline conjugates (26.89–27.48%). These results align with You et al. [35], who reported that free radical grafting of ovotransferrin with catechin led to marked M.W. band shifts, whereas no obvious change was observed in alkaline conjugates. However, less pronounced shifts have been reported for WPI conjugated with (-)-epigallocatechin-3-gallate (EGCG) using the free radical grafting method [18,31]. These findings emphasize that the conjugation method, polyphenol structure, and protein composition collectively determine the M.W. of the resulting conjugates.

#### 3.1.2. UV and FTIR Spectra

**Figure 2:**
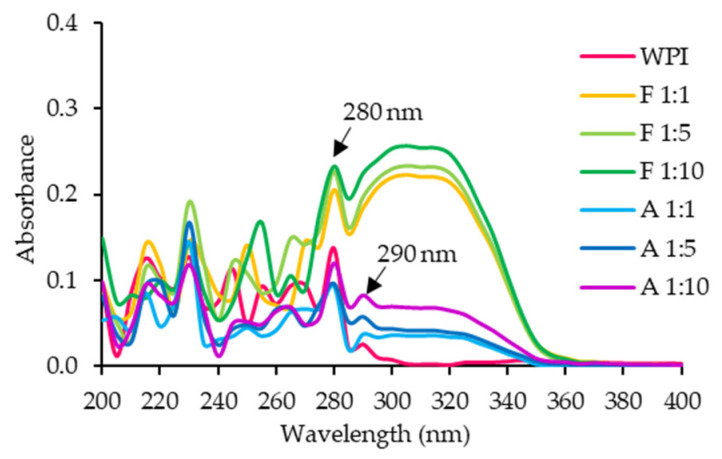
UV spectra of whey protein isolate (WPI) and WPI–resveratrol conjugates prepared using free radical grafting (F) and alkaline (A) methods at WPI/resveratrol ratios of 1:1, 1:5, and 1:10.

The UV spectrum of WPI (Figure 2) exhibited characteristic peaks at 214 nm (peptide bond absorption) and 280 nm (aromatic amino acid absorption) [10]. Conjugates from both methods showed a significant increase in absorbance at 280 nm and within the 300–320 nm range (Figure 2) compared to the untreated WPI, consistent with covalent bonding between resveratrol and WPI. An increase in absorbance at 305–316 nm signaled the presence of resveratrol moieties, with intensity increasing with the resveratrol concentration. Only the alkaline conjugates displayed an increase at 290 nm, which corresponds to the absorbance of tryptophan [36]. This may be linked to the mechanism of the alkaline method, in which phenolic compounds are oxidized into reactive quinones that subsequently interact with proteins [35]. The formation of these quinones likely enhances interactions with aromatic amino acids in WPI, potentially modifying peptide bonds and triggering protein structural rearrangements. To further elucidate the molecular basis of these interactions, future studies employing kinetic or thermodynamic modeling, fluorescence spectroscopy, as well as molecular simulations and docking, are recommended [29].

**Figure 3:**
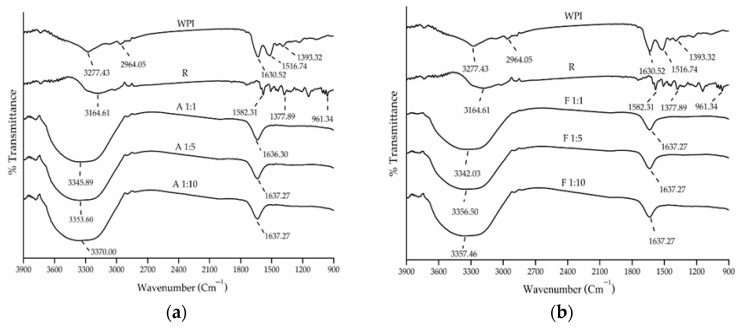
FTIR spectra of whey protein isolate (WPI), resveratrol (R), and WPI–R conjugates prepared using (**a**) alkaline method; (**b**) free radical grafting method, at WPI/R ratios of 1:1, 1:5, and 1:10.

Both conjugation methods induced a shift in the FTIR peaks in the amide A region (hydroxyl or N-H groups; 3277.43 cm^−1^) and in the amide I region (C=O stretching; 1630.52 cm^−1^) and resulted in the disappearance of the amide II peak (N-H bending and C-N stretching; 1516.74 cm^−1^) as seen in Figure 3. Increasing resveratrol concentrations further shifted the amide A peak, with alkaline conjugates (3345.89–3370.00 cm^−1^) being more affected than free radical grafting conjugates (3342.03–3357.46 cm^−1^). In contrast, the amide I peak showed minimal changes as resveratrol concentration varied. These spectral changes indicate interactions between resveratrol and the N-H, C-N, and COO- groups of WPI, revealing alterations in the secondary structure of WPI.

The secondary structure analysis (Table 1 and Figures S1–S3) showed a significant decrease in α-helix content and an increase in β-sheet content for both methods (*p* < 0.05), while the β-turn and random coil content remained unchanged (*p* > 0.05). No significant differences were observed between the alkaline and free radical grafting conjugates or among different resveratrol concentrations (*p* > 0.05). These results are consistent with previous reports on protein–polyphenol conjugates, where changes in secondary structure reflect covalent interactions and protein rearrangement [11,37]. Changes in the proportion and arrangement of α-helix, β-sheet, β-turn, and random coil dictate how a protein interacts with its environment and, consequently, its functional attributes [38].

#### 3.1.3. Total Phenolic Content and % Grafting Efficiency

The total phenolic content and grafting efficiency of the conjugates (Table 2) increased with higher resveratrol concentrations. Conjugates prepared via the free radical grafting method exhibited significantly higher total phenolic content and grafting efficiency (15.89–17.61%) than those obtained using the alkaline method (9.52–10.65%) (*p* < 0.05). The increase in grafting efficiency with rising resveratrol concentration was especially pronounced in the free radical grafting conjugates. This is likely due to the different bonding mechanisms involved.

Under alkaline conditions, polyphenols are readily oxidized to semiquinone radicals that form covalent bonds with proteins [39]. However, resveratrol instability above pH 6.8 leads to increased degradation [4,40], resulting in the lower efficiency observed with the alkaline method. In contrast, the free radical grafting method activates amino acids on the protein chains, promoting covalent bonding with the hydroxyl groups of resveratrol [17,41]. It may also induce partial protein unfolding, thereby increasing the accessibility of reactive amino acids and enhancing grafting efficiency. These findings align with the SDS-PAGE profiles (Figure 1a) and UV spectra (Figure 2) of the conjugates.

The free radical grafting conjugate at a 1:10 ratio exhibited the highest total phenolic content and grafting efficiency, representing a 3.61-fold and 1.65-fold increase, respectively, compared to alkaline conjugates at the same concentration. These results indicate that the free radical grafting method is more effective for loading resveratrol into WPI–resveratrol conjugates, which may be beneficial for functional food applications. Although additional reactive amino acid residues may remain available on WPI, precipitation of resveratrol at concentrations higher than the 1:10 ratio limited further increases in this study. Strategies that further promote protein unfolding may improve conjugation efficiency [11].

### 3.2. Solubility and Functional Properties

#### 3.2.1. Water Solubility

Solubility is a crucial property that influences the applicability of proteins and bioactive compounds in various industries, particularly in functional foods and beverages. Several factors, including pH, salt concentration, temperature, and interactions with other compounds in the matrix, influence protein solubility. Conjugating protein with polyphenol can either increase or decrease solubility, depending on the structural characteristics of the resulting conjugates [34,41]. Ordered structures, like α-helix and β-sheet are generally associated with reduced solubility and limited emulsifying and foaming properties. In contrast, disordered structures, such as β-turn and random coil are related to protein unfolding or rearrangement that may enhance functional properties [38].

**Figure 4:**
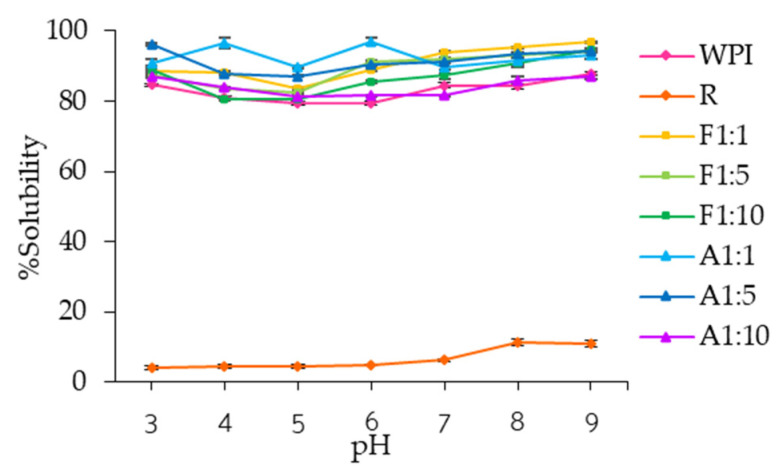
Water solubility of whey protein isolate (WPI), resveratrol (R), and WPI–R conjugates prepared using free radical grafting (F) and alkaline (A) methods at WPI/R ratios of 1:1, 1:5, and 1:10.

In this study, water solubility was determined for WPI, resveratrol, and WPI–resveratrol conjugates across pH 3–9 (Figure 4 and Table S2). WPI exhibited solubility ranging from 79.42% to 87.91% (7.94–8.79 mg/mL), with the lowest values observed at pH 5 (79.54 ± 0.57%, 7.95 mg/mL) and pH 6 (79.42 ± 0.70%, 7.94 mg/mL) (*p* > 0.05), likely due to aggregation near its isoelectric point (pH 4.8–6). In contrast, resveratrol showed significantly lower solubility across the tested pH range (*p* < 0.05), ranging from 4.10% (0.41 mg/mL) at pH 3 to a maximum of 11.23% (1.12 mg/mL) at pH 8. These results are consistent with previous studies, which report relatively constant resveratrol solubility between pH 3 and 6, and increased solubility above pH 7 [4,40]. However, according to Zupančič et al. [42], prolonged exposure to high pH may eventually decrease resveratrol solubility due to deprotonation and subsequent degradation.

WPI–resveratrol conjugates from both methods exhibited significantly higher water solubility (*p* < 0.05) than native WPI and resveratrol, with all conjugate values exceeding 80.5% (8.05 mg/mL). This enhancement is attributed to changes in the net charge of WPI after conjugation, resulting from protein unfolding, decreased surface hydrophobicity, and increased hydrophilicity and water compatibility [34]. Secondary structure analysis (Table 1) corroborates these changes through showing a decrease in α-helix content and an increase in β-turn and random coil structures, indicating unfolding and exposure of polar residues that promote hydrogen bonding and solubility. However, increasing resveratrol concentration reduced solubility, likely due to excessive crosslinking, which promotes aggregation and the formation of more complex molecular structures [41,43]. It also explains the higher solubility observed in alkaline conjugates compared to free radical grafting conjugates, which had higher grafting efficiency with resveratrol (Table 2). Overall, these results demonstrate that both conjugation methods are promising for incorporating compounds with poor water solubility, such as resveratrol, into aqueous food and beverage applications.

#### 3.2.2. Emulsifying Properties

**Figure 5:**
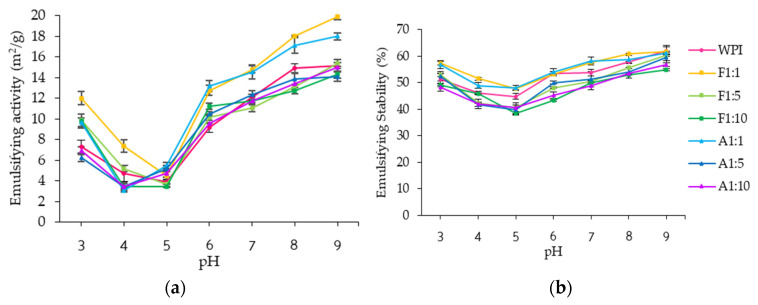
(**a**) Emulsifying activity; (**b**) emulsifying stability of whey protein isolate (WPI), and WPI–resveratrol conjugates prepared using free radical grafting (F) and alkaline (A) methods at WPI/resveratrol ratios of 1:1, 1:5, and 1:10.

Emulsifying properties refer to the ability of proteins to form and stabilize emulsions composed of two immiscible liquids, such as water and oil phases. Effective emulsifiers require amphiphilic surfaces capable of reducing interfacial tension between the two phases and preventing droplet flocculation or coalescence [11]. In this study, the emulsifying activity index (EAI) and emulsion stability index (ESI) of WPI and its conjugates were assessed as a function of pH and resveratrol concentration (Figure 5 and Tables S3 and S4).

Both conjugation methods improved the EAI of WPI. Conjugation with resveratrol promoted protein unfolding, exposing both hydrophilic and hydrophobic regions and reduced interfacial tension at the oil–water interface [44]. This increased interaction between the water and oil phases enhanced emulsification efficiency [17,34,41], consistent with secondary structure analyses that showed a decrease in α-helix content and an increase in β-sheet structure. The lowest EAI was observed at pH 5 for WPI (3.86 ± 0.28%) and free radical grafting conjugates (3.43–4.52%), while alkaline conjugates exhibited their lowest EAI at pH 4 (3.03–3.42%). This likely results from enhanced aggregation near the isoelectric point, which reduces solubility and interface flexibility [39]. The differences in EAI between conjugate methods are attributable to structural differences observed in the UV and FTIR spectra (Figure 2 and Figure 3).

At pH 3–4, free radical grafting conjugates showed significantly higher EAI than both WPI and alkaline conjugates (*p* < 0.05). At pH 6 and above, the EAI differences between the two conjugate methods at the same resveratrol concentrations were less pronounced (Figure 5a). As resveratrol concentration increased, the EAI of conjugates decreased, likely due to excessive crosslinking, resulting in reduced molecular flexibility and hindered adsorption at the oil–water interface [45]. Although both methods improved EAI, enhancement of the ESI was observed across pH 3–9 only when resveratrol was added at a 1:1 ratio (Figure 5b). Higher concentrations of resveratrol resulted in a decrease in conjugates’ ESI, likely because increased resveratrol content reduced electrostatic repulsion (possibly surface charge) between oil droplets, promoting aggregation and reducing emulsion stability [45]. These results suggest that adjustment of protein-to-resveratrol ratio and pH can optimize both the emulsifying activity and stability of the conjugates for specific food applications.

#### 3.2.3. Foaming Properties

**Figure 6:**
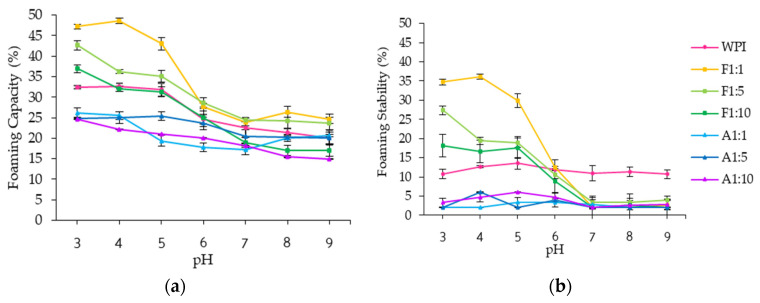
(**a**) Foaming capacity; (**b**) foaming stability of whey protein isolate (WPI), and WPI–resveratrol conjugates prepared using free radical grafting (F) and alkaline (A) methods at WPI/resveratrol ratios of 1:1, 1:5, and 1:10.

Foaming contributes to the desirable texture and structure of many food applications, including beverages, baked goods, confectionery, dairy, and frozen desserts [46]. The foaming capacity (FC) and foaming stability (FS) of WPI and its conjugates were influenced by pH and concentration of resveratrol (Figure 6 and Tables S5 and S6). The FC of WPI ranged from 20.12 ± 1.82% to 33.34 ± 1.35%, with the values decreasing as pH or resveratrol concentration increased.

Free radical grafting conjugates with 1:1 and 1:5 ratios exhibited significantly higher FC, ranging from 1.22- to 1.46-fold and 1.09- to 1.29-fold increases, respectively, compared to WPI across all studied pH values except at pH 7 (*p* < 0.05). Alkaline conjugates had the lowest FC (14.96–26.24%) for each pH and resveratrol concentration.

All free radical grafting conjugates also showed higher FS than WPI and alkaline conjugates at pH 5 and below, regardless of resveratrol concentration. Alkaline conjugates had significantly lower FS (2.03–6.08%) compared to WPI (10.73–13.53%). Overall, only free radical grafting conjugates demonstrated consistent improvements in both FC (31.36–48.61%) and FS (16.68–36.11%) over WPI within the pH range of 3–5. This enhancement is likely due to higher grafting efficiency of resveratrol and protein structural changes, such as unfolding and increased exposure of hydrophilic and hydrophobic groups, induced by the free radical grafting method, which promote rapid adsorption and stabilization at the air–water interface [34]. These modifications collectively facilitate efficient interfacial arrangement and adsorption, leading to improved foam formation and stability.

### 3.3. Antioxidant Activities

WPI exhibits antioxidant activities through multiple mechanisms, including radical scavenging activity, reducing power, and metal ion chelation [47]. It can also synergistically improve the antioxidant capacity of other compounds in food systems [48]. In this study, WPI exhibited DPPH (49.23 ± 1.54 µmol TE/g), ABTS (84.79 ± 3.49 µmol TE/g), and FRAP(28.35 ± 2.73 µmol TE/g) activities, indicating its capacity for radical scavenging and reducing power.

After conjugating with resveratrol, all antioxidant activities increased significantly (*p* < 0.05), with enhancements depending on both the conjugation method and resveratrol concentration (Table 3). Free radical grafting conjugates showed higher DPPH (1.3–2.1 fold), ABTS (2.5–3.6 fold), and FRAP (1.3–2.1 fold) values than alkaline conjugates, likely due to higher grafting efficiency and greater incorporation of resveratrol. These findings are consistent with previous studies reporting improved antioxidant activity following conjugation of WPI with polyphenols such as EGCG [18], proanthocyanidins [49], chlorogenic acid, quercetin, and rosmarinic acid [42]. Increasing the concentration of resveratrol further enhanced the antioxidant activity of the conjugates, likely by introducing additional hydroxyl groups that increase the electron- and hydrogen-donating capacity of the protein molecules [17,41].

Although unreacted resveratrol was thoroughly removed from all conjugate samples, the possibility remains that the observed increases in antioxidant activities may be influenced by additive effects. Inclusion of additional controls, such as physical mixtures of WPI and resveratrol, in future studies will be important to more definitively distinguish the effects of covalent conjugation from simple additive effects, thus providing a more comprehensive mechanistic understanding.

## 4. Conclusions

In this study, we demonstrated that the free radical grafting method is more effective than the alkaline method for conjugating WPI with resveratrol. This approach significantly enhanced resveratrol’s solubility, as well as the protein’s functional and antioxidant properties. Compared to the alkaline method at the same resveratrol concentration, free radical grafting produced conjugates with improved foaming and emulsifying capacities, as well as higher antioxidant activities across multiple assays. These findings confirm the potential of WPI–resveratrol conjugates as a promising strategy for incorporating resveratrol into functional food systems. Our results demonstrate that the conjugation method, structural properties of WPI, and the WPI-to-resveratrol ratio collectively play crucial roles in defining the molecular characteristics and functional properties of the resulting conjugates.

## Figures and Tables

**Table 1:** Secondary structure (%) of whey protein isolate (WPI) and WPI–resveratrol conjugates prepared using free radical grafting (F) and alkaline (A) methods at WPI/resveratrol ratios of 1:1, 1:5, and 1:10.

Sample	α-Helix	β-Sheet	β-Turn ^ns^	Random Coil ^ns^
WPI	15.73 ± 0.64 ^a^	58.00 ± 1.21 ^b^	23.05 ± 0.73	3.22 ± 1.10
A 1:1	13.46 ± 0.86 ^b^	60.72 ± 1.18 ^a^	22.62 ± 1.76	3.20 ± 1.42
A 1:5	13.82 ± 0.57 ^b^	60.46 ± 1.48 ^a^	21.97 ± 1.76	3.74 ± 0.81
A 1:10	13.94 ± 0.64 ^b^	60.22 ± 1.54 ^a^	22.56 ± 2.01	3.28 ± 1.11
F 1:1	13.46 ± 1.12 ^b^	60.62 ± 0.65 ^a^	22.39 ± 0.93	3.53 ± 1.07
F 1:5	13.63 ± 0.69 ^b^	60.60 ± 1.09 ^a^	23.01 ± 1.79	2.76 ± 1.40
F 1:10	13.89 ± 0.29 ^b^	60.03 ± 1.33 ^a^	22.51 ± 1.96	3.57 ± 1.07

Different letters in the same column indicate significant differences (*p* < 0.05); ns, not significant difference (*p* ≥ 0.05).

**Table 2:** Total phenolic content (mg gallic acid/g) and grafting efficiency (%) of WPI–resveratrol conjugates prepared using free radical grafting (F) and alkaline (A) methods at WPI/resveratrol ratios of 1:1, 1:5, and 1:10.

Sample	Total Phenolic Content	Grafting Efficiency
A 1:1	10.26 ± 1.06 ^f^	9.52 ± 0.91 ^e^
A 1:5	13.69 ± 0.94 ^e^	10.02 ± 0.63 ^de^
A 1:10	15.91 ± 0.63 ^d^	10.65 ± 0.38 ^d^
F 1:1	32.23 ± 1.50 ^c^	15.89 ± 0.92 ^c^
F 1:5	43.12 ± 1.01 ^b^	16.87 ± 0.31 ^b^
F 1:10	57.50 ± 1.58 ^a^	17.61 ± 0.67 ^a^

Different letters in the same column indicate significant differences (*p *< 0.05).

**Table 3:** Antioxidant activities (µmol Trolox equivalent/g) of whey protein isolate (WPI), resveratrol, and WPI–resveratrol conjugates prepared using free radical grafting (F) and alkaline (A) methods at WPI/resveratrol ratios of 1:1, 1:5, and 1:10.

Sample	DPPH	ABTS	FRAP
WPI	49.23 ± 1.54 ^h^	84.79 ± 3.49 ^h^	28.35 ± 2.73 ^h^
Resveratrol	435.16 ± 1.29 ^a^	667.52 ± 2.17 ^a^	459.21 ± 4.98 ^a^
A 1:1	86.37 ± 1.76 ^g^	87.93 ± 1.75 ^g^	91.59 ± 1.73 ^g^
A 1:5	92.48 ± 1.83 ^f^	114.06 ± 1.69 ^f^	146.83 ± 1.58 ^f^
A 1:10	144.32 ± 2.61 ^c^	122.63 ± 2.71 ^e^	173.42 ± 3.27 ^e^
F 1:1	110.78 ± 3.13 ^e^	246.48 ± 3.67 ^d^	142.63 ± 2.30 ^d^
F 1:5	139.12 ± 3.61 ^d^	285.30 ± 1.20 ^c^	190.62 ± 2.26 ^c^
F 1:10	297.99 ± 2.04 ^b^	438.59 ± 2.77 ^b^	360.99 ± 1.31 ^b^

Different letters in the same column indicate significant differences (*p *< 0.05).

## Data Availability

The original contributions presented in this study are included in the article/Supplementary Material. Further inquiries can be directed to the corresponding author.

## Supplementary Materials

The following supporting information can be downloaded at: https://www.mdpi.com/article/10.3390/foods14152596/s1, Table S1. Molecular weight distribution of whey protein isolate (WPI) and WPI-resveratrol conjugates prepared using free radical grafting (F) and alkaline (A) methods at WPI:resveratrol ratios of 1:1, 1:5, and 1:10; Table S2: Water solubility of whey protein isolate (WPI), resveratrol (R), and WPI-R conjugates prepared using free radical grafting (F) and alkaline (A) methods at WPI:R ratios of 1:1, 1:5, and 1:10; Table S3: Emulsifying activity index of whey protein isolate (WPI) and WPI-resveratrol conjugates prepared using free radical grafting (F) and alkaline (A) methods at WPI:resveratrol ratios of 1:1, 1:5, and 1:10; Table S4: Emulsion stability index of whey protein isolate (WPI) and WPI-resveratrol conjugates prepared using free radical grafting (F) and alkaline (A) methods at WPI:resveratrol ratios of 1:1, 1:5, and 1:10; Table S5. Foaming Capacity of whey protein isolate (WPI) and WPI-resveratrol conjugates prepared using free radical grafting (F) and alkaline (A) methods at WPI:resveratrol ratios of 1:1, 1:5, and 1:10; Table S6: Foaming Stability of whey protein isolate (WPI) and WPI-resveratrol conjugates prepared using free radical grafting (F) and alkaline (A) methods at WPI:resveratrol ratios of 1:1, 1:5, and 1:10; Figure S1. Amide I band fitting curve of whey protein isolate (WPI); Figure S2. Amide I band fitting curve of whey protein isolate (WPI)-resveratrol conjugates prepared using free radical grafting (F) method at WPI:resveratrol ratios of 1:1, 1:5, and 1:10; Figure S3. Amide I band fitting curve of whey protein isolate (WPI)-resveratrol conjugates prepared using alkaline (A) method at WPI:resveratrol ratios of 1:1, 1:5, and 1:10.

## Abbreviations

The following abbreviations are used in this manuscript:

WPI	Whey protein isolate
R	Resveratrol
F	Free radical grafting method
A	Alkaline method
DPPH	2,2-Diphenyl-1-picrylhydrazyl
ABTS	2,2′-Azinobis(3-ethylbenzothiazoline-6-sulfonic acid)
FRAP	Ferric reducing antioxidant power
