# Supplementary material for: Comparative Study of Free Radical Grafting and Alkaline Conjugation for Enhanced Resveratrol Incorporation and Whey Protein Functionalities
Source: Foods. 2025 Jul 24;14(15):2596. doi: 10.3390/foods14152596 (PMC12346753; doi:10.3390/foods14152596)
Supplement: Supplementary file 1 [file foods-14-02596-s001.zip › foods-3759226-supplementary.pdf]

Table S1. Molecular weight distribution of whey protein isolate (WPI) and WPI-resveratrol conjugates prepared using free radical grafting (F) and alkaline (A) methods at WPI:resveratrol ratios of 1:1, 1:5, and 1:10.

Sample	Molecular weight (Da)				
	>100,000	10,001-100,000	1,001-10,000	101-1,000^{ns}	<100
WPI	0.64 ± 0.00^{a}	26.03 ± 0.02^{d}	68.62 ± 0.51^{ab}	3.85 ± 1.56	0.85 ± 0.08^{a}
F 1:1	0.26 ± 0.03^{c}	27.12 ± 0.11^{cd}	68.17 ± 1.61^{ab}	4.15 ± 1.37	0.31 ± 0.15^{c}
F 1:5	0.36 ± 0.01^{b}	28.52 ± 0.71^{ab}	67.50 ± 0.70^{ab}	3.14 ± 0.12	0.47 ± 0.11^{bc}
F 1:10	0.41 ± 0.03^{b}	29.14 ± 0.15^{a}	66.63 ± 0.54^{b}	3.08 ± 0.83	0.74 ± 0.12^{ab}
A 1:1	0.24 ± 0.06^{c}	26.89 ± 0.35^{cd}	69.26 ± 0.25^{a}	3.37 ± 0.22	0.25 ± 0.07^{c}
A 1:5	0.29 ± 0.04^{c}	27.29 ± 1.00^{bcd}	68.53 ± 0.61^{ab}	3.60 ± 1.78	0.30 ± 0.14^{c}
A 1:10	0.36 ± 0.06^{b}	27.48 ± 0.28^{bc}	67.73 ± 0.99^{ab}	3.79 ± 0.62	0.64 ± 0.04^{ab}

Different letters in the same column indicate significant differences (P<0.05); ns, not significant difference (P>0.05).

Table S2. Water solubility of whey protein isolate (WPI), resveratrol (R), and WPI-R conjugates prepared using free radical grafting (F) and alkaline (A) methods at WPI:R ratios of 1:1, 1:5, and 1:10.

pH	Sample							
	WPI	Resveratrol	F 1:1	F 1:5	F 1:10	A 1:1	A 1:5	A 1:10
3	87.91 ± 0.46^{hi}	4.10 ± 0.47^{u}	88.36 ± 0.77^{ghi}	87.26 ± 0.80^{ij}	89.07 ± 0.99^{fgh}	96.69 ± 1.55^{a}	96.04 ± 1.01^{ab}	86.91 ± 1.72^{j}
4	80.99 ± 0.40^{p}	4.45 ± 0.42^{u}	88.10 ± 1.36^{ghi}	83.66 ± 0.65^{m}	80.66 ± 0.50^{pq}	90.70 ± 1.13^{ef}	87.82 ± 1.30^{hi}	84.00 ± 1.00^{kl}
5	79.54 ± 0.57^{qr}	4.50 ± 0.53^{u}	83.59 ± 1.73^{m}	82.56 ± 1.21^{n}	80.52 ± 0.92^{pq}	89.70 ± 0.51^{fg}	87.17 ± 1.05^{hij}	81.19 ± 0.73^{op}
6	79.42 ± 0.70^{qr}	4.80 ± 0.15^{u}	91.23 ± 1.07^{de}	88.97 ± 2.33^{gh}	85.44 ± 0.49^{jk}	89.73 ± 2.08^{fg}	90.36 ± 1.18^{f}	81.72 ± 1.60^{no}
7	84.48 ± 0.71^{k}	6.23 ± 0.49^{t}	91.75 ± 1.49^{de}	91.06 ± 0.95^{de}	81.58 ± 1.43^{nop}	94.02 ± 1.00^{bcd}	91.92 ± 1.40^{de}	87.49 ± 1.31^{hij}
8	84.36 ± 1.14^{k}	11.23 ± 0.84^{s}	93.09 ± 0.98^{cde}	93.44 ± 1.18^{cd}	85.90 ± 0.66^{jk}	95.44 ± 1.20^{abc}	93.05 ± 0.99^{cde}	90.97 ± 1.43^{ef}
9	84.59 ± 0.36^{k}	10.78 ± 095^{s}	97.03 ± 0.99^{a}	94.42 ± 1.17^{bc}	86.85 ± 0.38^{j}	96.73 ± 1.57^{a}	94.15 ± 1.05^{bc}	94.81 ± 0.37^{bc}

Different letters indicate significant differences (P<0.05).

Table S3. Emulsifying activity index of whey protein isolate (WPI) and WPI-resveratrol conjugates prepared using free radical grafting (F) and alkaline (A) methods at WPI:resveratrol ratios of 1:1, 1:5, and 1:10.

pH	Sample						
	WPI	F 1:1	F 1:5	F 1:10	A 1:1	A 1:5	A 1:10
3	7.28 ± 0.66^{k}	11.99 ± 0.63^{fg}	9.82 ± 0.68^{ij}	9.78 ± 0.35^{ij}	9.66 ± 0.38^{ij}	6.25 ± 0.42^{l}	6.93 ± 0.37^{kl}
4	4.71 ± 0.80^{n}	7.35 ± 0.60^{k}	5.15 ± 0.30^{m}	3.46 ± 0.27^{op}	3.03 ± 0.15^{p}	3.44 ± 0.35^{op}	3.42 ± 0.06^{op}
5	3.86 ± 0.28^{o}	4.52 ± 0.46^{n}	3.67 ± 0.04^{o}	3.43 ± 0.11^{op}	5.44 ± 0.34^{m}	5.16 ± 0.29^{m}	4.75 ± 0.39^{n}
6	9.15 ± 0.49^{j}	12.74 ± 0.51^{ef}	10.15 ± 0.30^{hi}	11.24 ± 0.67^{gh}	13.18 ± 0.51^{e}	10.49 ± 0.17^{h}	9.58 ± 0.05^{ij}
7	11.50 ± 0.53^{g}	14.79 ± 0.34^{c}	11.09 ± 0.36^{gh}	11.75 ± 0.38^{g}	14.51 ± 0.68^{cd}	12.31 ± 0.43^{f}	11.75 ± 0.13^{g}
8	14.89 ± 0.41^{c}	18.02 ± 0.09^{ab}	13.07 ± 0.29^{e}	12.70 ± 0.26^{ef}	17.11 ± 0.77^{b}	13.87 ± 0.58^{de}	13.40 ± 0.98^{e}
9	15.17 ± 0.33^{c}	19.90 ± 0.33^{a}	15.39 ± 0.36^{c}	14.29 ± 0.25^{cde}	17.97 ± 0.32^{ab}	14.10 ± 0.44^{cde}	15.02 ± 0.43^{c}

Different letters indicate significant differences (P<0.05).

Table S4. Emulsion stability index of whey protein isolate (WPI) and WPI-resveratrol conjugates prepared using free radical grafting (F) and alkaline (A) methods at WPI:resveratrol ratios of 1:1, 1:5, and 1:10.

pH	Sample						
	WPI	F 1:1	F 1:5	F 1:10	A 1:1	A 1:5	A 1:10
3	51.19 ± 2.23^{i}	57.25 ± 1.37^{ef}	52.84 ± 1.52^{hi}	49.07 ± 2.38^{l}	56.69 ± 1.46^{f}	52.33 ± 1.85^{ij}	48.19 ± 1.30^{lm}
4	46.17 ± 0.67^{o}	51.47 ± 1.85^{j}	42.31 ± 1.39^{r}	45.83 ± 1.92^{op}	48.90 ± 1.04^{l}	41.69 ± 0.74^{rs}	41.91 ± 1.73^{rs}
5	44.81 ± 1.10^{pq}	47.68 ± 1.63^{n}	40.70 ± 1.52^{t}	38.32 ± 2.99^{tu}	47.96 ± 0.99^{n}	39.70 ± 1.85^{tu}	40.66 ± 1.63^{t}
6	53.53 ± 0.50^{gh}	53.12 ± 1.24^{h}	48.03 ± 1.79^{lm}	43.30 ± 4.20^{q}	54.08 ± 1.12^{gh}	49.98 ± 10.60^{kl}	45.19 ± 1.30^{p}
7	53.70 ± 1.29^{gh}	57.53 ± 1.27^{e}	50.43 ± 1.63^{k}	50.52 ± 2.49^{k}	58.11 ± 1.51^{de}	51.23 ± 2.82^{j}	48.93 ± 1.53^{l}
8	57.73 ± 0.40^{e}	60.87 ± 2.86^{b}	55.54 ± 2.18^{g}	52.98 ± 1.80^{hi}	58.73 ± 1.48^{d}	54.10 ± 1.21^{g}	53.53 ± 1.80^{h}
9	61.93 ± 1.60^{a}	61.61 ± 1.07^{a}	60.63 ± 1.95^{b}	54.79 ± 2.48^{g}	61.16 ± 2.89^{a}	59.63 ± 2.13^{c}	56.76 ± 0.78^{f}

Different letters indicate significant differences ($P<0.05$).

Table S5. Foaming Capacity of whey protein isolate (WPI) and WPI-resveratrol conjugates prepared using free radical grafting (F) and alkaline (A) methods at WPI:resveratrol ratios of 1:1, 1:5, and 1:10.

pH	Sample						
	WPI	F 1:1	F 1:5	F 1:10	A 1:1	A 1:5	A 1:10
3	33.12 ± 1.41^{d}	47.22 ± 0.61^{a}	42.67 ± 1.15^{b}	36.91 ± 1.01^{c}	26.24 ± 1.23^{fgh}	24.83 ± 0.29^{hij}	24.67 ± 0.02^{hij}
4	33.34 ± 1.35^{d}	48.61 ± 0.60^{a}	36.24 ± 0.42^{c}	32.01 ± 0.64^{d}	25.51 ± 0.88^{ghi}	25.01 ± 1.34^{ghi}	22.15 ± 0.07^{klm}
5	31.78 ± 1.68^{d}	43.07 ± 1.52^{b}	35.14 ± 1.42^{c}	31.36 ± 1.18^{d}	19.32 ± 1.33^{pq}	25.34 ± 1.06^{fghi}	20.97 ± 0.02^{mn}
6	22.62 ± 2.30^{kl}	27.59 ± 1.02^{ef}	28.67 ± 1.15^{e}	24.99 ± 2.22^{ghi}	17.80 ± 0.98^{rs}	23.67 ± 1.53^{jk}	20.00 ± 0.10^{op}
7	24.67 ± 1.15^{hij}	23.97 ± 0.91^{jk}	24.50 ± 0.50^{hij}	18.93 ± 1.30^{q}	17.25 ± 1.31^{rst}	20.39 ± 1.63^{no}	18.24 ± 0.04^{qr}
8	21.47 ± 0.92^{lm}	26.40 ± 1.33^{fg}	24.31 ± 1.78^{ij}	17.01 ± 1.22^{rst}	20.13 ± 0.88^{no}	20.27 ± 0.65^{no}	15.43 ± 0.07^{u}
9	20.12 ± 1.82^{no}	24.67 ± 1.15^{hij}	23.67 ± 1.53^{jk}	17.03 ± 1.50^{rst}	20.69 ± 0.25^{mno}	20.01 ± 1.42^{op}	14.96 ± 0.02^{uv}

Different letters indicate significant differences ($P<0.05$).

Table S6. Foaming Stability of whey protein isolate (WPI) and WPI-resveratrol conjugates prepared using free radical grafting (F) and alkaline (A) methods at WPI:resveratrol ratios of 1:1, 1:5, and 1:10.

pH	Sample						
	WPI	F 1:1	F 1:5	F 1:10	A 1:1	A 1:5	A 1:10
3	10.82 ± 1.24^{hi}	34.72 ± 0.69^{a}	27.33 ± 1.15^{c}	18.12 ± 2.01^{def}	2.13 ± 0.00^{n}	2.07 ± 0.02^{n}	3.33 ± 1.15^{lmn}
4	12.66 ± 0.93^{gh}	36.11 ± 0.66^{a}	19.46 ± 0.94^{d}	16.68 ± 1.47^{f}	2.07 ± 0.02^{n}	6.08 ± 0.07^{k}	4.71 ± 1.23^{kl}
5	13.53 ± 1.35^{g}	29.88 ± 1.78^{b}	18.93 ± 1.30^{de}	17.66 ± 0.59^{ef}	3.46 ± 1.23^{lmn}	2.05 ± 0.02^{n}	6.08 ± 0.07^{k}
6	12.00 ± 2.00^{ghi}	12.42 ± 0.30^{ghi}	10.67 ± 1.15^{i}	8.79 ± 1.23^{j}	3.43 ± 1.20^{lmn}	4.06 ± 0.10^{lmn}	4.67 ± 1.15^{klm}
7	10.97 ± 2.43^{hi}	2.05 ± 0.02^{n}	3.40 ± 1.18^{lmn}	2.03 ± 0.02^{n}	2.76 ± 1.22^{lmn}	2.04 ± 0.04^{n}	2.03 ± 0.02^{n}
8	11.41 ± 1.23^{hi}	2.08 ± 0.04^{n}	3.37 ± 1.16^{lmn}	2.72 ± 1.18^{mn}	2.08 ± 0.04^{n}	2.10 ± 0.07^{n}	2.69 ± 1.20^{mn}
9	10.73 ± 1.10^{i}	2.67 ± 1.15^{n}	4.06 ± 0.10^{lmn}	2.04 ± 0.04^{n}	2.07 ± 0.02^{n}	2.07 ± 0.02^{n}	2.74 ± 1.24^{mn}

Different letters indicate significant differences ($P<0.05$).

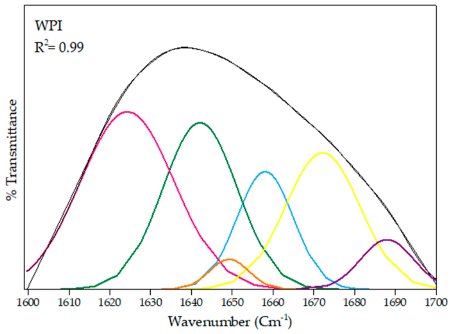

Figure S1. Amide I band fitting curve of whey protein isolate (WPI)

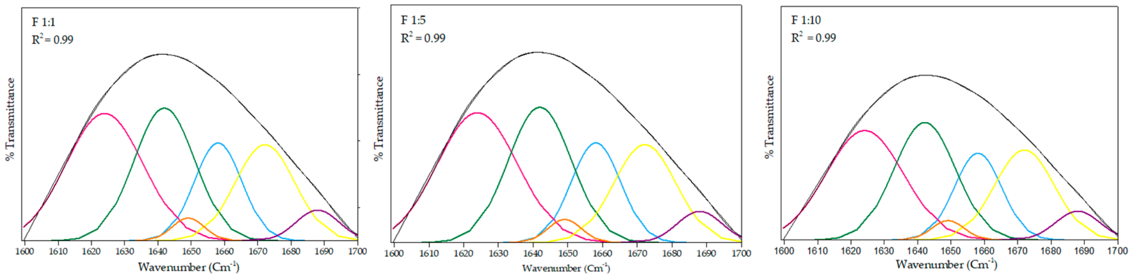

Figure S2. Amide I band fitting curve of whey protein isolate (WPI)-resveratrol conjugates prepared using free radical grafting (F) method at WPI:resveratrol ratios of 1:1, 1:5, and 1:10.

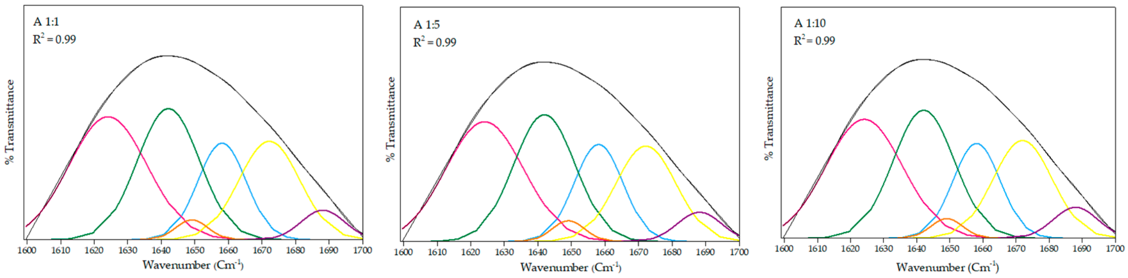

Figure S3. Amide I band fitting curve of whey protein isolate (WPI)-resveratrol conjugates prepared using alkaline (A) method at WPI:resveratrol ratios of 1:1, 1:5, and 1:10.
